# Exploring the Role of Persuasive Design in Unguided Internet-Delivered Cognitive Behavioral Therapy for Depression and Anxiety Among Adults: Systematic Review, Meta-analysis, and Meta-regression

**DOI:** 10.2196/26939

**Published:** 2021-04-29

**Authors:** Hugh C McCall, Heather D Hadjistavropoulos, Christopher Richard Francis Sundström

**Affiliations:** 1 Department of Psychology University of Regina Regina, SK Canada; 2 PSPNET University of Regina Regina, SK Canada; 3 Department of Clinical Neuroscience Centre for Psychiatry Research Karolinska Institutet Stockholm Sweden; 4 Department of Psychology Stockholm University Stockholm Sweden

**Keywords:** ICBT, internet, depression, anxiety, persuasive design, eHealth

## Abstract

**Background:**

Internet-delivered cognitive behavioral therapy (ICBT) is an effective treatment that can overcome barriers to mental health care. Various research groups have suggested that unguided ICBT (ie, ICBT without therapist support) and other eHealth interventions can be designed to enhance user engagement and thus outcomes. The persuasive systems design framework captures most design recommendations for eHealth interventions, but there is little empirical evidence that persuasive design is related to clinical outcomes in unguided ICBT.

**Objective:**

This study aims to provide an updated meta-analysis of randomized controlled trials of unguided ICBT for depression and anxiety, describe the frequency with which various persuasive design principles are used in such interventions, and use meta-regression to explore whether a greater number of persuasive design elements predicts efficacy in unguided ICBT for depression and anxiety.

**Methods:**

We conducted a systematic review of 5 databases to identify randomized controlled trials of unguided ICBT for depression and anxiety. We conducted separate random effects meta-analyses and separate meta-regressions for depression and anxiety interventions. Each meta-regression included 2 steps. The first step included, as a predictor, whether each intervention was transdiagnostic. For the meta-regression of ICBT for depression, the first step also included the type of control condition. The number of persuasive design principles identified for each intervention was added as a predictor in the second step to reveal the additional variance in effect sizes explained by persuasive design.

**Results:**

Of the 4471 articles we identified in our search, 46 (1.03%) were eligible for inclusion in our analyses. Our meta-analyses showed effect sizes (Hedges *g*) ranging from 0.22 to 0.31 for depression interventions, depending on the measures taken to account for bias in the results. We found a mean effect size of 0.45 (95% CI 0.33-0.56) for anxiety interventions, with no evidence that the results were inflated by bias. Included interventions were identified as using between 1 and 13 persuasive design principles, with an average of 4.95 (SD 2.85). The meta-regressions showed that a greater number of persuasive design principles predicted greater efficacy in ICBT for depression (*R*^2^ change=0.27; *B*=0.04; *P*=.02) but not anxiety (*R*^2^ change=0.05; *B*=0.03; *P*=.17).

**Conclusions:**

These findings show wide variability in the use of persuasive design in unguided ICBT for depression and anxiety and provide preliminary support for the proposition that more persuasively designed interventions are more efficacious, at least in the treatment of depression. Further research is needed to clarify the role of persuasive design in ICBT.

## Introduction

### Background

Depression and anxiety are highly prevalent and represent the leading and the sixth leading causes of disability worldwide, respectively [1]. Despite the demonstrated efficacy of psychotherapeutic and pharmacological interventions for depression and anxiety [2-4], many people face structural barriers to accessing mental health care (eg, financial barriers, transportation barriers, inconvenience, and limited availability of services) [5,6]. Internet-delivered cognitive behavioral therapy (ICBT) is the most common type of internet intervention and an effective treatment for several common mental health problems, including depression and anxiety [7]. Unlike traditional cognitive behavioral therapy (CBT), ICBT enables users to access treatment materials privately at a time and location that is convenient for them, allowing it to be administered economically on a large scale and circumvent barriers to traditional forms of mental health care [8-10]. ICBT can be therapist guided or unguided. Guidance appears to improve adherence and clinical outcomes [11], but unguided ICBT is economical, highly scalable, and believed by many researchers to have considerable potential for improving public health [12-15].

Since the early 2000s, various research groups have suggested that eHealth interventions such as unguided ICBT can be designed in ways that improve user engagement and thus outcomes. In 2003, Fogg [16] presented the *functional triad* principle, suggesting that technology can function as a tool, a medium for relaying content, and a social actor to help facilitate behavior change. In 2009, Oinas-Kukkonen and Harjumaa [17] developed the *persuasive systems design* (PSD) framework, which elaborated on the functional triad and included 28 recommended design principles to produce more persuasive and engaging technological systems. They divided these principles into 4 categories: (1) *primary task support* principles, which facilitate the completion of the primary tasks of an intervention or other system; (2) *dialogue support* principles, through which an intervention or other system supports a user to help them enact their target behavior; (3) *system credibility support* principles, which facilitate a more credible and persuasive intervention or other system; and (4) *social support* principles, which leverage principles of social psychology to help users of an intervention or other system motivate one another. The 28 principles are described in Multimedia Appendix 1 [17].

Several other research groups have provided their own design recommendations for eHealth interventions. Despite using different terminology, most of these recommendations appear to align closely with the principles included in the PSD framework. Examples include recommendations related to *personalization* [18-22], *tailoring* [19,21,22], *reminders* [19,20], *self-monitoring* [18,23], *liking* [19,22,24], and various *dialogue support* principles [18,19,23]. A few design recommendations are not captured in the PSD framework (eg, time-limited access [20] and greater use of metaphors [22]), but to our knowledge, none of these have been proposed by 2 or more research groups; that is, the PSD framework appears to capture most common recommendations. Various groups’ recommendations and the related PSD framework principles are displayed in Multimedia Appendix 2 [18-24].

In 2012, Kelders et al [25] used the PSD framework to assess whether the persuasive design principles used in 83 eHealth interventions for chronic conditions, lifestyle changes, and mental health predicted adherence. They conducted a meta-regression, finding that a greater number of dialogue support principles predicted greater adherence to eHealth interventions. However, to our knowledge, there is no empirical research demonstrating a relationship between persuasive design and symptom change in eHealth interventions.

### Objectives and Hypothesis

This study aims to (1) present a systematic review and meta-analysis of randomized controlled trials of unguided ICBT for depression and anxiety among adults, (2) systematically examine the frequency with which various persuasive design principles are used in such interventions, and (3) use meta-regression to examine the extent to which persuasive design could explain the variability in effect sizes identified through the meta-analysis. Thus, the overarching objective of this study is to review the efficacy, the use of persuasive design, and the relationship between efficacy and persuasive design in unguided ICBT for depression and anxiety. We hypothesized that using a greater number of persuasive design principles would predict greater efficacy among the included studies.

## Methods

### Study Design

This study consisted of a systematic review, 2 meta-analyses, and 2 meta-regressions. The methods used in each phase of the study are described in the following sections. We registered the methodological protocol for this study on PROSPERO on October 24, 2019 (ID: 153466), before commencing the literature search, and kept a log of revisions to the original protocol throughout the course of this research (Multimedia Appendix 3 [26-28]). We followed the guidelines outlined in the PRISMA (Preferred Reporting Items for Systematic Reviews and Meta-Analyses) statement in the preparation of this paper [29].

### Systematic Review Methods

#### Eligibility Criteria

We searched for randomized controlled trials of unguided ICBT interventions for symptoms of depression and/or anxiety among adults that had been published in English in academic journals since 2000. We included trials of ICBT targeting symptoms of any type of depressive or anxiety disorder, as defined in the fifth edition of the Diagnostic and Statistical Manual of Mental Disorders [30], with various kinds of control conditions (eg, waitlist, treatment as usual, and active control). Studies involving samples with a mean age of less than 18 years were excluded.

Although we excluded studies in which ICBT was delivered with guidance from a therapist or coach, we did not exclude studies involving diagnostic interviews or contact of a logistical nature between participants and research teams. Interventions that used a CBT model of treatment and were delivered via the internet were considered ICBT interventions regardless of whether the authors of trials identified them as such. We included interventions using third-wave CBT approaches (eg, mindfulness-based CBT and acceptance and commitment therapy) [31] because prior research has not demonstrated significant differences in outcomes between traditional CBT and third-wave approaches [32,33].

#### Literature Search

On October 29, 2019, we conducted a literature search on MEDLINE, PsycINFO, PubMed, Web of Science, and PsycArticles. To be identified, articles were required to include the words “CBT,” “internet,” “trial,” and “depression” or “anxiety” or one of several similar phrases for each of these terms in their titles, keywords, or abstracts. The search terms are shown in detail in Multimedia Appendix 4. This search was updated on July 2, 2020.

#### Study Selection

After removing duplicates of studies identified in 2 or more databases, HCM and CRFS independently screened the studies in 3 stages: by title, by abstract, and by full text. Wherever the 2 screeners reached different decisions about whether to retain or exclude a study, that study was included in the next stage of screening. Differences in decisions on the full-text screening were resolved through discussion.

#### Data Extraction

HCM extracted several types of data from each study: study characteristics (eg, type of control condition and time between pretreatment and posttreatment measures), risk of bias [34], general intervention characteristics (eg, target symptoms and medium of delivery), persuasive design principles [17] as operationalized by Kelders et al [25], and efficacy data. Consistent with the approach of Kelders et al [25], we did not code principles in the *system credibility support* category of the PSD framework because they were reported very infrequently and would have been challenging to code objectively (eg, a system should have a “competent look and feel” and “provide endorsements from respected sources” [17]). In most cases, we coded persuasive design principles as present or absent based on the descriptions of interventions in the included studies, although we consulted other available sources of information when possible (eg, intervention websites and study protocols). The complete list of data items is provided in Multimedia Appendix 5. The persuasive design principle *tunneling*, which refers to the sequential presentation of treatment elements in a structured, linear manner, was not counted toward the total number of persuasive design principles in this study. This is because researchers have recently proposed that eHealth interventions can be made more engaging by providing users with greater flexibility and control concerning the modules or features they wish to use [19,22], which contrasts with the principle of *tunneling*.

#### Risk of Bias Assessment

We assessed the risk of bias among included studies using the Cochrane risk of bias tool [34]. We did not assess the risk domain *blinding participants and personnel* because it is not possible for participants to be blind to their conditions in psychotherapy research [35]. Furthermore, we did not assess the risk domain *blinding of outcome assessment* because all outcome measures were self-report measures, and participants could thus not be blinded. Self-report measures are generally considered equivalent to blind clinical observers in psychotherapy research, and research suggests that they do not result in inflated effect sizes [35].

### Meta-analysis Methods

We conducted meta-analyses using Comprehensive Meta-Analysis software (Biostat Inc) [36]. As prior research suggests that ICBT for generalized and social anxiety is more efficacious than ICBT for depression [7,37], we conducted separate meta-analyses of ICBT for anxiety and ICBT for depression. Given the availability of symptom change data for both anxiety and depression, trials of ICBT designed to treat both conditions were included in both meta-analyses. We measured heterogeneity in the effect sizes of the included studies using the *I*^2^ index and formally tested the degree of heterogeneity using the *Q* statistic [38]. In each of the 2 meta-analyses, we used a random effects model, used between-groups effect size (Hedges *g*) as the summary measure, and weighted each study by the inverse of the within-study variance of the primary outcome measure plus the between-study variance. Several studies evaluated 2 unguided ICBT interventions; in such cases, we treated the evaluation of each intervention as a separate study, except we divided the control group sample size by 2, such that each control group participant was included only once in the analyses [39]. We evaluated the risk of publication bias using funnel plots and accounted for publication bias using the trim and fill technique [40]. We explored the influence of study-level bias on outcomes by repeating the meta-analyses without studies deemed to be at high risk on one or more dimensions of the Cochrane tool for assessing risk of bias [34].

### Meta-regression Methods

We conducted 2 meta-regressions using Comprehensive Meta-Analysis [36]—one for depression interventions and one for anxiety interventions—to determine the degree to which persuasive design principles could explain variance in effect sizes among studies. Paralleling the approach taken to the meta-analyses, we included trials of ICBT designed to treat both depression and anxiety in both meta-regressions, given the availability of symptom change data for both conditions. We also weighted each study by the inverse of the within-study variance of the primary outcome measure plus the between-study variance, as in the meta-analyses.

We used 3 predictor variables. Our main predictor of interest was the total number of persuasive design principles identified for each intervention. We were unable to include the number of persuasive design principles in each category of the PSD framework as separate predictors, as Kelders et al [25] did, because of the risk of overfitting, given the limited number of included studies. We also input a binary variable reflecting whether each intervention was transdiagnostic (ie, designed to treat symptoms of both depression and anxiety). We did this to account for the possibility that unguided ICBT focused on treating a narrower range of symptoms (ie, anxiety or depression) may be more efficacious for treating those symptoms than transdiagnostic unguided ICBT designed to treat a broader range of symptoms (ie, both depression and anxiety). Our final predictor was a binary variable reflecting whether each study used a control condition with active elements (eg, psychoeducation and mood monitoring) because a previous meta-analysis of unguided ICBT found a large mean effect size among studies using passive control conditions and a small mean effect size among studies using active control conditions [41]. However, the control condition type was not included as a predictor in the meta-regression of ICBT for anxiety because there were insufficient studies to justify an additional predictor variable (eg, because of the risk of overfitting), following most recommendations concerning acceptable *subjects per variable* ratios in linear regression analyses [26].

We conducted each meta-regression in 2 steps. The first step included transdiagnostic status and, for the meta-regression of ICBT for depression, the control condition type. In both meta-regressions, the number of persuasive design principles identified was then added in the second step. This 2-step approach was used to reveal the amount of additional variance persuasive design explained in the second step after accounting for the other variables in the first step.

We conducted 5 assumption tests at each step of each meta-regression. First, we examined Pearson *r* correlations and scatterplots to test the assumption of linearity of the relationship between each continuous predictor variable and Hedges *g* [42]. Second, we checked Cook distance values to identify any outlier studies that had unduly large influences on the results [43]. Third, we inspected the distribution of studentized residuals using a histogram to ensure that the residuals were normally distributed [42]. Fourth, we inspected scatterplots plotting studentized residuals against predicted values to test the assumption of homoscedasticity [42]. Finally, we examined variance inflation factors to check for multicollinearity [42].

## Results

### Systematic Review Results

#### Study Selection

**Figure 1:**
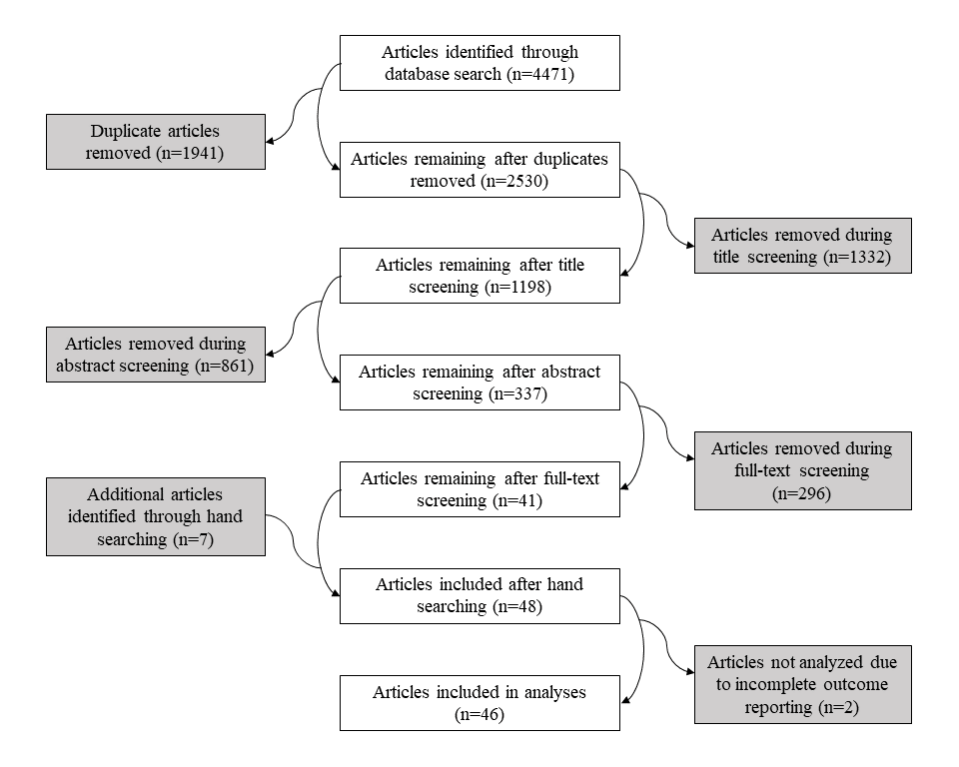
Flow of studies through the study selection process.

Between the original and updated literature searches, we identified 4471 articles, 39 of which were found eligible for analysis. Having found another 7 eligible articles through a hand search, we included a total of 46 articles. The flow of studies through the study selection process is shown in Figure 1. Separate flowcharts for the original and updated literature searches are shown in Multimedia Appendices 6 and 7, respectively. The 2 screeners (HCM and CRFS) made the same screening decision (ie, retain or remove) for 81.66% (2066/2530) of articles during the title screening, 86.39% (1035/1198) of articles during the abstract screening, and 81.9% (276/337) of articles during the full-text screening.

#### Study Characteristics

The 46 eligible studies included 16,632 participants, excluding participants assigned to experimental groups irrelevant to this study (eg, guided ICBT groups). Studies were most often published in or after 2017 (24/46, 52%); included samples drawn from the general population (26/46, 57%), clinical populations (14/46, 30%), or both (6/46, 13%); and most often used waitlist control conditions without active elements (28/46, 61%). The characteristics of each study are presented in Table 1.

**Table 1:** Study characteristics.

Category and study		Intervention	Participant, n^a^	Duration in weeks^b,c^	Control condition	Recruitment population	
						Clinical	Nonclinical
**ICBT^d^ for depression**							
	Berger et al, 2011 [44]	Deprexis	51	10	Waitlist		✓
	Bücker et al, 2019 [45]	MOOD	125	6	Care as usual	✓	✓
	Clarke et al, 2002 [46]	ODIN^e^	299	4	Health information website	✓	
	Clarke et al, 2005 [47]	ODIN	175	5	Health information website	✓	
	Clarke et al, 2009 [48]	—^f^	160	16	Health information website	✓	
	Dahne et al, 2019 [49]	¡Aptívate!	33	8	Care as usual	✓	✓
	Dahne et al, 2019 [49]	iCouch CBT	20	8	Care as usual	✓	✓
	Dahne et al, 2019 [50]	Moodivate	33	8	Care as usual	✓	
	Dahne et al, 2019 [50]	MoodKit	28	8	Care as usual	✓	
	de Graaf et al, 2009 [51]	Colour Your Life	203	13.05	Care as usual		✓
	Farrer et al, 2011 [52]	MoodGym and Bluepages	73	6	Care as usual	✓	
	Gräfe et al, 2020 [53]	Deprexis	3805	12	Brochure and care as usual	✓	
	Hur et al, 2018 [54]	Todac Todac^g^	34	3	Mood charting app	✓	✓
	Lintvedt et al, 2013 [55]	MoodGym and Bluepages	163	8	Waitlist		✓
	Löbner et al, 2018 [56]	MoodGym (German adapted, version III)	647	6	Care as usual	✓	
	Lüdtke et al, 2018 [57]	Be good to yourself	88	4	Waitlist	✓	✓
	Lüdtke et al, 2018 [58]	—	132	4	Care as usual	✓	
	McDermott and Dozois, 2019 [59]	MoodGym	302	8	Attentional control		✓
	Meyer et al, 2009 [60]	Deprexis	396	9	Waitlist		✓
	Meyer et al, 2015 [61]	Deprexis	163	13.05	Waitlist	✓	✓
	Mira et al, 2017 [62]	Sonreír es Divertido^h^	80	12	Waitlist		✓
	Mohr et al, 2013 [63]	moodManager	68	6	Waitlist	✓	
	Montero-Marin et al, 2016 [64]	Smiling is Fun	124	13.05	Improved treatment as usual	✓	
	Moritz et al, 2012 [65]	Deprexis	210	8	Waitlist		✓
	Morris et al, 2015 [66]	Panoply	166	3	Web-based expressive writing		✓
	Noguchi et al, 2017 [67]	—	651	5	Waitlist		✓
	Schure et al, 2019 [68]	Thrive	343	8	Depression information website		✓
	Silverstone et al, 2017 [69]	MoodGym	109	12	Care as usual	✓	
	Spek et al, 2007 [70]	—	202	10	Waitlist		✓
**ICBT for depression and anxiety**							
	Bakker et al, 2018 [71]	MoodKit	120	4.29	Waitlist		✓
	Bakker et al, 2018 [71]	MoodMission	114	4.29	Waitlist		✓
	Kleiboer et al, 2015 [72]	Allesondercontrole^i^	213	6	Waitlist and web-based information		✓
	Moberg et al, 2019 [73]	Pacifica	500	4.35	Waitlist		✓
	Powell et al, 2013 [74]	MoodGym	3070	6	Waitlist		✓
	Proudfoot et al, 2013 [75]	myCompass	459	8	Waitlist		✓
	Shirotsuki et al, 2017 [76]	—	48	6	Mood monitoring		✓
	Twomey et al, 2014 [77]	MoodGym	66	4.57	Waitlist	✓	
**ICBT for anxiety**							
	Berger et al, 2017 [78]	Velibra	139	9	Waitlist	✓	
	Boettcher et al, 2018 [79]	Challenger	139	7	Waitlist		✓
	Boettcher et al, 2018 [79]	—	139	7	Waitlist		✓
	Botella et al, 2010 [80]	Talk to Me	91	8.7	Waitlist		✓
	Ciuca et al, 2018 [81]	PAXPD^j^	75	12	Waitlist	✓	✓
	Donker et al, 2019 [82]	0Phobia	193	3	Waitlist		✓
	Ivanova et al, 2016 [83]	Ångesthjälpen^k^	102	10	Waitlist		✓
	Kenardy et al, 2003 [84]	—	83	6	Waitlist		✓
	Lin et al, 2020 [85]	—	26	8	Waitlist		✓
	McCall et al, 2018 [86]	Overcome Social Anxiety	101	17.4	Waitlist		✓
	Oh et al, 2020 [87]	Todaki	41	4	Book on panic disorder	✓	
	Powell et al, 2020 [88]	E-couch	2116	6	Waitlist		✓
	Titov et al, 2008 [89]	Shyness	64	10	Waitlist		✓

^a^For the purpose of this table, n was calculated as the number of participants assigned to the intervention identified in each row plus the number of participants assigned to the control condition (ie, excluding participants assigned to use other interventions).

^b^Study duration expressed in days was divided by 7. Study duration expressed in months was multiplied by 4.35 (the average number of weeks in a month during a 365-day year).

^c^Some studies reported data from multiple posttreatment time points; for such studies, the duration, as shown in this table, is the number of weeks between pretreatment and whichever posttreatment time point was selected for use in the analyses reported in this study.

^d^ICBT: internet-delivered cognitive behavioral therapy.

^e^ODIN: Overcoming Depression on the Internet.

^f^Data were not reported.

^g^Todac Todac translates to “Tap Tap.”

^h^Sonreír es Divertido translates to “Smiling is Fun.”

^i^Allesondercontrole translates to “all is under control.”

^j^PAXPD: PAXonline Program for Panic Disorder.

^k^Ångesthjälpen translates to “The Anxiety Help.”

#### Risk of Bias

We evaluated the risk of bias among included studies using 5 of the 7 domains in the Cochrane risk of bias tool [34]. Of the 46 included studies, 14 (30%) were identified to be at high risk of bias in at least one domain, whereas only 4 (9%) were found to be at low risk of bias in all domains assessed. Most studies (28/46, 61%) were found to be at low or unclear risk in each domain. The risk of bias identified in each study is presented in Table 2.

**Table 2:** Risk of bias in included studies.

Category and study			Random sequence generation		Allocation concealment		Incomplete outcome data (attrition bias)		Selective reporting		Other bias
**ICBT^a^ for depression**											
	Berger et al, 2011 [44]	Low		Unclear		Low		Unclear		Low	
	Bücker et al, 2019 [45]	Low		Low		Low		Unclear		Low	
	Clarke et al, 2002 [46]	Low		Low		Low		Unclear		Low	
	Clarke et al, 2005 [47]	Low		Low		High		Unclear		Low	
	Clarke et al, 2009 [48]	Unclear		Unclear		Low		Unclear		Unclear	
	Dahne et al, 2019 [49]	Unclear		Unclear		Low		Unclear		Low	
	Dahne et al, 2019 [50]	Unclear		Unclear		Unclear		Unclear		Low	
	de Graaf et al, 2009 [51]	Low		Low		Low		Low		Low	
	Farrer et al, 2011 [52]	Unclear		Low		Low		Unclear		Low	
	Gräfe et al, 2020 [53]	Low		Unclear		Low		Low		Low	
	Hur et al, 2018 [54]	Low		Low		Low		Unclear		Low	
	Lintvedt et al, 2013 [55]	Low		Unclear		Low		Unclear		Low	
	Löbner et al, 2018 [56]	Low		Low		Low		Unclear		Low	
	Lüdtke et al, 2018 [57]	Unclear		Low		Low		Unclear		Low	
	Lüdtke et al, 2018 [58]	High		Low		Low		Unclear		Low	
	McDermott and Dozois, 2019 [59]	Unclear		Unclear		Low		Unclear		Unclear	
	Meyer et al, 2009 [60]	Low		High		High		Unclear		Low	
	Meyer et al, 2015 [61]	Low		Low		Low		Unclear		Low	
	Mira et al, 2017 [62]	Low		Low		Low		Unclear		Low	
	Mohr et al, 2013 [63]	Low		Low		Low		Unclear		Low	
	Montero-Marin et al, 2016 [64]	Low		Low		Low		Low		Low	
	Moritz et al, 2012 [65]	Unclear		Unclear		Low		Unclear		Low	
	Morris et al, 2015 [66]	Unclear		Unclear		Low		Unclear		Low	
	Noguchi et al, 2017 [67]	Low		Low		Low		Unclear		Low	
	Schure et al, 2019 [68]	Unclear		Unclear		Low		Low		Low	
	Silverstone et al, 2017 [69]	High		High		High		Unclear		Low	
	Spek et al, 2007 [70]	Unclear		Low		Low		Unclear		Low	
**ICBT for depression and anxiety**											
	Bakker et al, 2018 [71]	High		High		High		Unclear		Low	
	Kleiboer et al, 2015 [72]	Low		Low		High		Unclear		Unclear	
	Moberg et al, 2019 [73]	Unclear		Unclear		High		Unclear		Low	
	Powell et al, 2013 [74]	Low		Low		Low		Low		Low	
	Proudfoot et al, 2013 [75]	Low		Low		High		Unclear		Low	
	Shirotsuki et al, 2017 [76]	Unclear		Low		Low		Unclear		Low	
	Twomey et al, 2014 [77]	Low		High		High		Unclear		Low	
**ICBT for anxiety**											
	Berger et al, 2017 [78]	Low		Low		Low		Unclear		Low	
	Boettcher et al, 2018 [79]	Low		Low		Low		Unclear		Low	
	Botella et al, 2010 [80]	Unclear		Unclear		High		Unclear		Unclear	
	Ciuca et al, 2018 [81]	Low		Low		Low		Unclear		Low	
	Donker et al, 2019 [82]	Low		Low		Low		Low		Low	
	Ivanova et al, 2016 [83]	Low		Low		High		Low		Low	
	Kenardy et al, 2003 [84]	Unclear		Unclear		Low		Unclear		Unclear	
	Lin et al, 2020 [85]	Low		Unclear		High		Unclear		Unclear	
	McCall et al, 2018 [86]	Low		High		Low		Unclear		Low	
	Oh et al, 2020 [87]	Unclear		Unclear		Low		Low		Low	
	Powell et al, 2020 [88]	Low		Low		High		Low		Low	
	Titov et al, 2008 [89]	Low		Unclear		Low		Unclear		Low	

^a^ICBT: internet-delivered cognitive behavioral therapy.

#### Intervention Characteristics

In total, 37 unguided ICBT interventions were evaluated in the 46 included studies. Of these 37 interventions, 15 (41%) were designed to treat depression exclusively and 9 (24%) were designed to treat depression and anxiety or stress. Other interventions were designed to treat social anxiety (6/37, 16%), panic (2/37, 5%), fear of public speaking (1/37, 3%), generalized anxiety (1/37, 3%), acrophobia (1/37, 3%), or symptoms of multiple anxiety disorders (2/37, 5%). Most interventions (23/37, 62%) were described as traditional CBT interventions, but many interventions (9/37, 24%) were described as being strongly influenced by elements of third-wave CBT (eg, mindfulness) or other therapeutic approaches (eg, positive psychology), and several interventions were based on behavioral activation (2/37, 5%), cognitive therapy (2/37, 5%), or problem-solving therapy (1/37, 3%). Half of the interventions (19/37, 51%) were delivered via a web browser, but many interventions were delivered via a mobile app (11/37, 30%) or both a browser and an app (5/37, 14%). Of the 37 interventions, it was unclear how 2 (5%) interventions were delivered. The characteristics of each intervention are presented in Table 3.

**Table 3:** Intervention characteristics.

Study	Name of the intervention	Target symptoms	Theoretical approach	Composition	Delivery medium	Number of persuasive design principles identified
Dahne et al, 2019 [49]	¡Aptívate!	Depression	Behavioral activation	Unclear	Mobile app	8
Donker et al, 2019 [82]	0Phobia	Acrophobia	CBT^a^	6 modules	Mobile app	6
Kleiboer et al, 2015 [72]	Allesondercontrole^b^	Depression and anxiety	Problem-solving therapy	5 lessons	Web browser	1
Ivanova et al, 2016 [83]	Ångesthjälpen^c^	Panic and social anxiety	Acceptance and commitment therapy	8 modules	App, browser, and CD	4
Lüdtke et al, 2018 [57]	Be Good to Yourself	Depression	CBT with third-wave elements	4 modules	Mobile app	4
Boettcher et al, 2018 [79]	Challenger	Social anxiety	CBT	N/A^d^	Mobile app	13
de Graaf et al, 2009 [51]	Colour Your Life	Depression	CBT	9 modules	Web browser	2
Berger et al, 2011 [44]; Gräfe et al, 2020 [53]; Meyer et al, 2009 [60]; Meyer et al, 2015 [61]; and Moritz et al, 2012 [65]	Deprexis	Depression	CBT and other approaches	12 modules	Web browser	5
Powell et al, 2020 [88]	E-couch	Social anxiety	CBT	6 modules	App and browser	2
Dahne et al, 2019 [49]	iCouch CBT	Depression and anxiety	CBT	Unclear	Mobile app	1
Bücker et al, 2019 [45]	MOOD	Depression	CBT with third-wave elements	9 modules	Web browser	5
Farrer et al, 2011 [52]; Lintvedt et al, 2013 [55]; Löbner et al, 2018 [56]; McDermott and Dozois, 2019 [59]; and Twomey et al, 2014 [77]	MoodGym^e^	Depression and anxiety	CBT and other approaches	5 modules	Web browser	1
Dahne et al, 2019 [50]	Moodivate	Depression	Behavioral activation	7 modules	Mobile app	8
Bakker et al, 2018 [71] and Dahne et al, 2019 [50]	MoodKit	Depression and anxiety	CBT	4 features	Mobile app	5
Mohr et al, 2013 [63]	moodManager	Depression	CBT	18 lessons	Web browser	4
Bakker et al, 2018 [71]	MoodMission	Depression and anxiety	CBT	N/A	Mobile app	4
Proudfoot et al, 2013 [75]	myCompass	Depression, anxiety, and stress	CBT and other approaches	12 modules	App and browser	6
McCall et al, 2018 [86]	Overcome Social Anxiety	Social anxiety	CBT	7 modules	Web browser	8
Clarke et al, 2002 [46] and Clarke et al, 2005 [47]	ODIN^f^	Depression	Cognitive therapy	7 modules	Web browser	5
Moberg et al, 2019 [73]	Pacifica	Depression, anxiety, and stress	CBT and other approaches	Unclear	Mobile app	8
Morris et al, 2015 [66]	Panoply	Depression	Cognitive therapy	N/A	Web browser	8
Ciuca et al, 2018 [81]	PAXPD^g^	Panic disorder	CBT	16 modules	Web browser	3
Titov et al, 2008 [89]	Shyness	Social anxiety	CBT	6 lessons	Web browser	8
Mira et al, 2017 [62] and Montero-Marin et al, 2016 [64]	Sonreír es Divertido^h^	Depression	CBT and other approaches	10 modules	Web browser	5
Botella et al, 2010 [80]	Talk to Me	Fear of public speaking	CBT	Unclear	Web browser	6
Schure et al, 2019 [68]	Thrive	Depression	CBT	3 modules	App and browser	3
Hur et al, 2018 [54]	Todac Todac^i^	Depression	CBT	3 modules	Mobile app	7
Oh et al, 2020 [87]	Todaki	Panic	CBT	4 modes	Mobile app	7
Berger et al, 2017 [78]	Velibra	Various anxiety disorders	CBT and other approaches	6 sessions	Web browser	5
Boettcher et al, 2018 [79]	Not reported	Social anxiety disorder	CBT	9 modules	Unclear	2
Clarke et al, 2009 [48]	Not reported	Depression	CBT	4 sections	Web browser	8
Kenardy et al, 2003 [84]	Not reported	Anxiety	CBT	6 sessions	Web browser	3
Lin et al, 2020 [85]	Not reported	Social anxiety	CBT	8 modules	Web browser	10
Lüdtke et al, 2018 [58]	Not reported	Depression	CBT	1 module	App and browser	3
Noguchi et al, 2017 [67]	Not reported	Depression and stress	CBT	Unclear	Web browser	1
Shirotsuki et al, 2017 [76]	Not reported	Depression- and anxiety-related symptoms	CBT	6 modules	e-learning system and guidebook	2
Spek et al, 2007 [70]	Not reported	Depression	CBT	8 modules	Web browser	2

^a^CBT: cognitive behavioral therapy.

^b^Allesondercontrole translates to “all is under control.”

^c^Ångesthjälpen translates to “The Anxiety Help.”

^d^N/A: not applicable.

^e^Bluepages was offered as a complement to MoodGym in studies by Farrer et al [52] and Lintvedt et al [55] but was omitted from this table (and all analyses) because it is a psychoeducation package and not an internet-delivered cognitive behavioral therapy intervention.

^f^ODIN: Overcoming Depression on the Internet.

^g^PAXPD: PAXonline Program for Panic Disorder.

^h^Sonreír es Divertido translates to “Smiling is Fun.”

^i^Todac Todac translates to “Tap Tap.”

#### Persuasive Design

On average, interventions included 4.95 (SD 2.85) persuasive design principles (excluding *tunneling*). The total number of persuasive design elements ranged from 1 to 13. Principles in the *primary task support* category were the most common (mean 2.86, SD 1.32), followed by principles in the *dialogue support* category (mean 1.27, SD 1.19) and *social support* category (mean 0.81, SD 1.60). The number of interventions in which each persuasive design principle was identified is presented in Table 4.

**Table 4:** Persuasive design principles identified.

Persuasive design principle			Brief description^a^		Interventions used, n (%)
**Primary task support**					
	Reduction	Divides target behavior into simple steps		35 (95)	
	Tunneling	Delivers content in a step-by-step format		29 (78)	
	Tailoring	Provides content adapted to user group		2 (5)	
	Personalization	Provides content that is adapted to one user		18 (49)	
	Self-monitoring	Provides ability to monitor progress or status		20 (54)	
	Simulation	Provides ability to observe relevant behavior		6 (16)	
	Rehearsal	Provides ability to rehearse a behavior		25 (68)	
**Dialogue support**					
	Praise	Offers praise to participant		8 (22)	
	Rewards	Offers reward to participant		5 (14)	
	Reminders	Provides reminders		13 (35)	
	Suggestion	Provides suggestions		15 (41)	
	Similarity	Is designed to look familiar		0 (0)	
	Liking	Is visually designed to be attractive		1 (3)	
	Social role	Acts as if it has a social role		5 (14)	
**Social support**					
	Social learning	Facilitates learning from other users		7 (19)	
	Social comparison	Facilitates comparison with other users		5 (14)	
	Normative influence	Provides normative information on target behavior		2 (5)	
	Social facilitation	Facilitates awareness of others using intervention		5 (14)	
	Cooperation	Stimulates users to cooperate		8 (22)	
	Competition	Stimulates users to compete		0 (0)	
	Recognition	Shows users who adopted target behavior		3 (8)	

^a^These descriptions were adapted from the operational definitions provided by Kelders et al [25].

### Meta-analysis Results

#### Meta-analysis of Unguided ICBT for Depression

**Figure 2:**
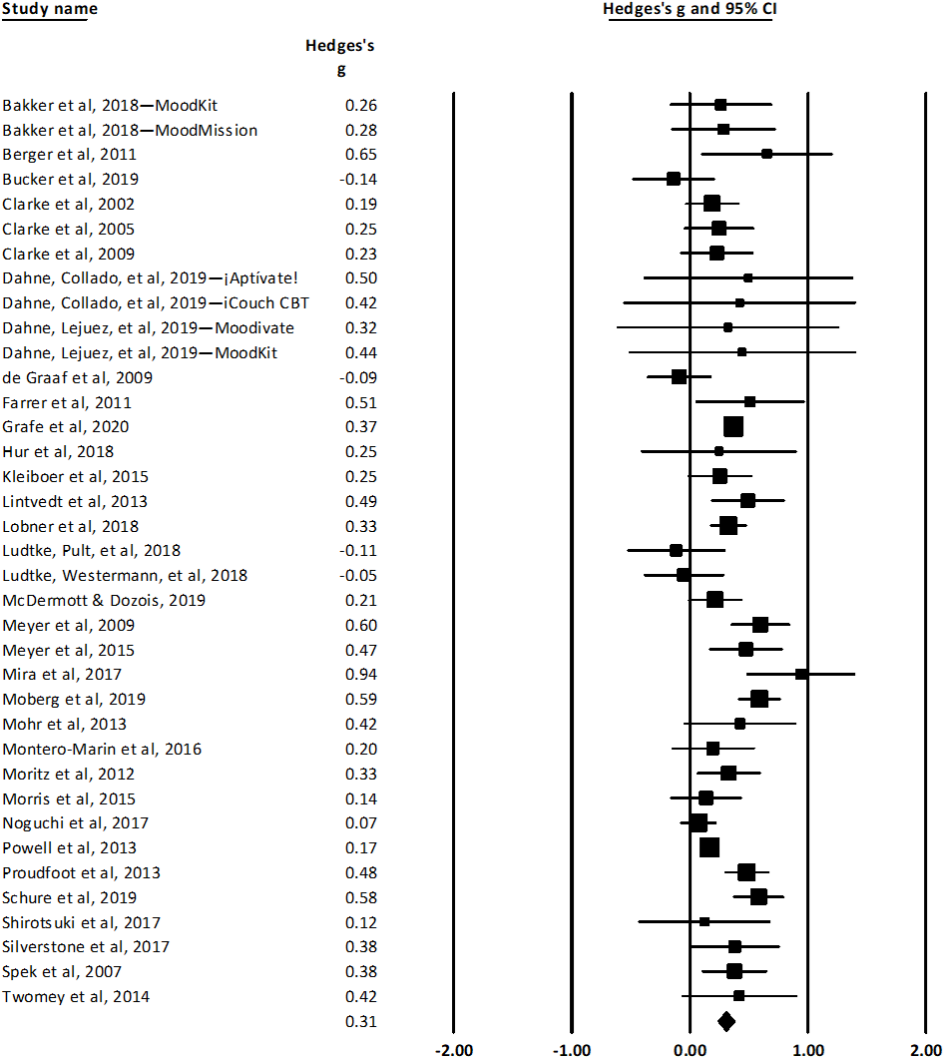
Meta-analysis of unguided internet-delivered cognitive behavioral therapy for depression.

We conducted a meta-analysis of 37 comparisons across 34 trials of unguided ICBT for depression. There was statistically significant heterogeneity in Hedges *g* among the studies (*Q*=89.85, *df*=36; *P*<.001). An *I*^2^ statistic of 59.93 indicated that a moderate proportion of variability was attributable to true heterogeneity rather than sampling error [90,91]. The weighted mean between-subjects effect size was small to moderate (Hedges *g*=0.31; SE 0.04; 95% CI 0.24-0.38). The forest plot for this meta-analysis is shown in Figure 2. Weighted mean effect sizes after excluding studies deemed to be at high risk of bias and after adjusting for publication bias using the trim and fill technique [40] are presented in Table 5.

**Table 5:** Summary statistics of meta-analyses with and without bias corrections.

Meta-analysis			Hedges *g* (95% CI)
**Meta-analysis of ICBT^a^ for depression**			
	All studies of ICBT for depression	0.31 (0.24-0.38)	
	All studies with trim and fill adjustment	0.23 (0.16-0.31)	
	Studies with high risk of bias excluded	0.28 (0.20-0.36)	
	Studies with high risk of bias excluded, with trim and fill adjustment	0.22 (0.14-0.31)	
**Meta-analysis** **of ICBT for anxiety**			
	All studies of ICBT for anxiety	0.45 (0.33-0.56)	
	All studies with trim and fill adjustment	0.45 (0.33-0.56)	
	Studies with high risk of bias excluded	0.54 (0.29-0.79)	
	Studies with high risk of bias excluded, with trim and fill adjustment	0.54 (0.29-0.79)	

^a^ICBT: internet-delivered cognitive behavioral therapy.

#### Meta-analysis of Unguided ICBT for Anxiety

**Figure 3:**
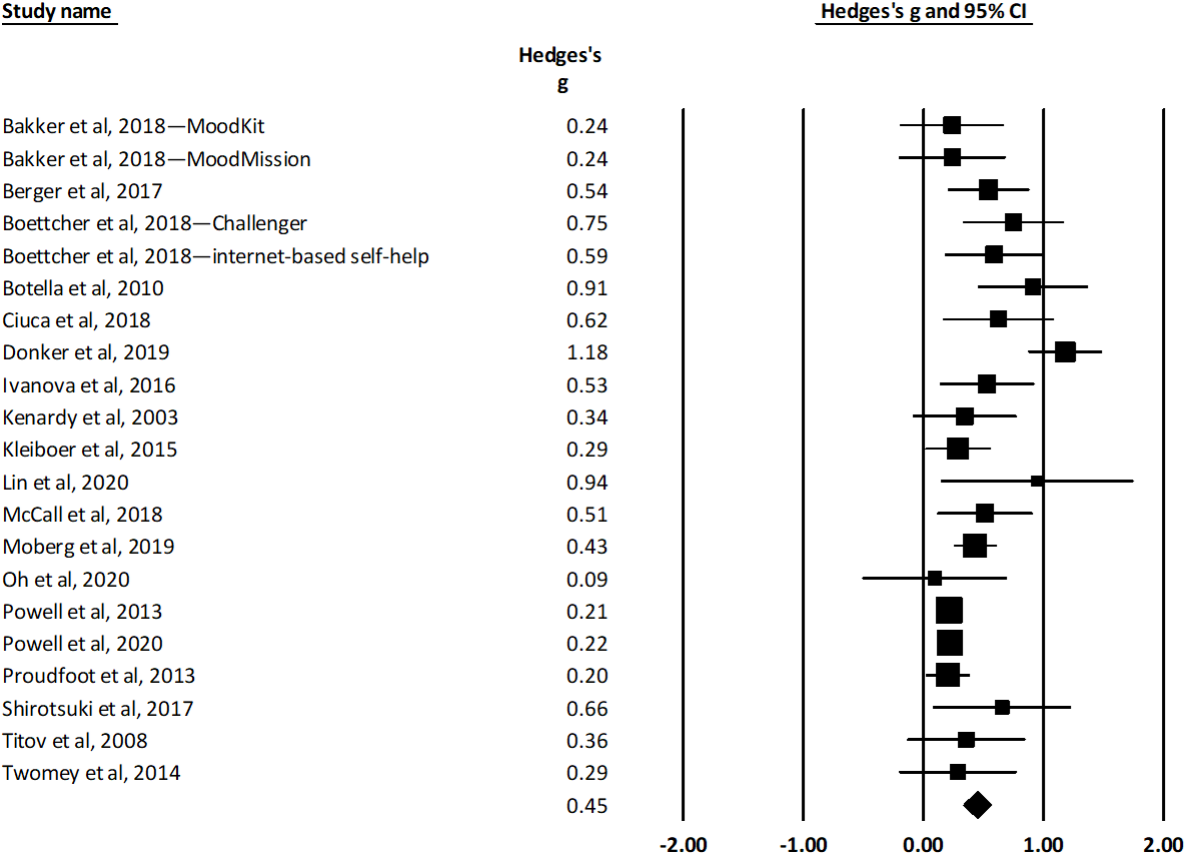
Meta-analysis of unguided internet-delivered cognitive behavioral therapy for anxiety.

We included 19 studies that reported 21 comparisons in a meta-analysis of unguided ICBT for anxiety. The results indicated statistically significant heterogeneity of Hedges *g* among studies (*Q*=68.47, *df*=20; *P*<.001). The corresponding *I*^2^ statistic of 70.79 suggested that a substantial proportion of the variability represented true heterogeneity [90,91]. The weighted mean between-subjects effect size was moderate (Hedges *g*=0.45; SE 0.06; 95% CI 0.33-0.56). A forest plot is shown in Figure 3. Additional weighted mean effect sizes accounting for publication- and study-level bias are presented in Table 5.

### Meta-regression Results

#### Meta-regression of Unguided ICBT for Depression

The meta-regression of ICBT for depression, like the meta-analysis of ICBT for depression, included 34 studies reporting 37 comparisons. We used 3 predictors in this meta-regression: the total number of persuasive design principles (mean 3.90, SD 2.33), whether each intervention was designed to treat symptoms of both depression and anxiety (19/37, 51%) or only depression (18/37, 49%), and whether each study used an active control condition (13/37, 35%) or a passive control condition (24/37, 65%). The results for both steps of the meta-regression are presented in Table 6. With the possible exception of very minor heteroscedasicity of residuals at one or both steps, all assumptions were met, as detailed in Multimedia Appendix 8.

**Table 6:** Meta-regression of unguided internet-delivered cognitive behavioral therapy for depression.

Step and variable			Model											Predictors												
			Model summary					*R* ^2^			*R*^2^ change			*B* ^a^			SE			95% CI			*P* value			Variance inflation factor
			*Q* (*df*)		*P* value																					
**Step 1**			1.05 (2)		.59		0.0			N/A^b^																
	Constant											0.28			0.07			0.15 to 0.41			<.001			3.03		
	Active control condition											−0.02			0.08			−0.19 to 0.14			.79			1.11		
	Transdiagnostic intervention											0.07			0.08			−0.09 to 0.23			.40			1.11		
**Step 2**			6.74 (3)		.08		0.27			0.27																
	Constant											0.10			0.10			−0.09 to 0.29			.32			8.63		
	Active control condition											−0.01			0.07			−0.16 to 0.13			.85			1.14		
	Transdiagnostic intervention											0.13			0.08			−0.02 to 0.28			.09			1.36		
	Persuasive design principles											0.04			0.02			0.01 to 0.07			.02			1.22		

^a^Unstandardized β coefficient.

^b^N/A: not applicable.

#### Meta-regression of Unguided ICBT for Anxiety

Similar to the meta-analysis of ICBT for anxiety, the meta-regression of ICBT for anxiety included 19 studies reporting 21 comparisons. We used 2 predictors: the total number of persuasive design principles (mean 5.05, SD 3.17) and whether each intervention was designed to treat symptoms of both depression and anxiety (8/21, 38%) or only anxiety (13/21, 62%). The results for both steps of the meta-regression are presented in Table 7. The assumption of normality of residuals may not have been met fully at both steps, although the residuals roughly approximated normal distributions. The assumption of homoscedasticity of the residuals was violated in step 1. The assumption tests for this meta-regression are detailed in Multimedia Appendix 9.

**Table 7:** Meta-regression of unguided internet-delivered cognitive behavioral therapy for anxiety.

Step and variable			Model								Predictors								
			Model summary				*R* ^2^		*R*^2^ change		*B* ^a^		SE		95% CI		*P* value		Variance inflation factor
			*Q* (*df*)		*P* value														
**Step 1**			4.80 (1)		.03		0.0		N/A^b^										
	Constant									0.57		0.08		0.41 to 0.73		<.001		1.77	
	Transdiagnostic intervention									−0.27		0.12		−0.51 to −0.03		.03		1.00	
**Step 2**			7.55 (2)		.02		0.05		0.05										
	Constant									0.42		0.13		0.18 to 0.67		<.001		5.12	
	Transdiagnostic intervention									−0.23		0.12		−0.46 to −0.00		.049		1.07	
	Persuasive design principles									0.03		0.02		−0.01 to 0.06		.17		1.07	

^a^Unstandardized β coefficient.

^b^N/A: not applicable.

## Discussion

### Principal Findings

Recent years have witnessed a proliferation of randomized trials of eHealth interventions, including many trials of unguided ICBT for depression and anxiety. Indeed, most of the studies included in this review were published in or after 2017. There was considerable diversity in the design of both studies (eg, study duration and type of control condition) and interventions (eg, mode of delivery and use of persuasive design principles).

The results of the meta-analysis of unguided ICBT for depression were consistent with the results of previous meta-analyses. We reported 4 mean effect sizes (Hedges *g*) for unguided ICBT for depression, ranging from 0.22 to 0.31, based on the corrections we made for publication bias and study-level bias. Previous meta-analyses of unguided ICBT for depression have found comparable mean effect sizes (Hedges *g* or Cohen *d*) ranging from 0.24 to 0.36 [12,92-95]. Our meta-analysis of unguided ICBT for anxiety yielded a mean effect size of 0.45. There was no evidence of publication bias, and the mean effect size was greater (Hedges *g*=0.54) after excluding studies found to be at a high risk of bias. Several previous meta-analyses of ICBT for symptoms of anxiety disorders found effect sizes between 0.70 and 1.12 [41,96-98]; however, all these meta-analyses included trials of guided ICBT interventions, which likely explains the greater mean effect sizes, at least in part. We are aware of only 1 meta-analysis that has included a subgroup analysis of *unguided* ICBT for anxiety—social anxiety, specifically—finding mean effect sizes (Hedges *g*) of 0.78 and 0.19 for studies using passive and active control conditions, respectively [41]. It is worth noting that our review included many transdiagnostic interventions designed to treat symptoms of both depression and anxiety. The meta-regressions showed that these interventions were significantly less efficacious for treating anxiety symptoms compared with interventions designed to treat anxiety symptoms only; however, their efficacy in treating depression did not significantly differ from interventions designed to treat symptoms of depression only.

We identified wide variability in the use of persuasive design in unguided ICBT for depression and anxiety, with several interventions using only 1 persuasive design principle and others using as many as 13. The intervention identified as having the greatest number of persuasive design principles (ie, 13), called *Challenger*, was specifically designed to be engaging, with many features inspired by the literature on gamification [79,99]. The mean number of persuasive design principles identified across interventions (4.95, excluding the principle of *tunneling*) was comparable with the mean of 5.4 principles identified by Kelders et al [25] among mental health interventions in their review. The mean number of persuasive design principles identified in the primary task support (mean 2.86, SD 1.32; excluding tunneling), dialogue support (mean 1.27, SD 1.19), and social support (mean 0.81, SD 1.60) categories were also roughly comparable with the corresponding means identified among mental health interventions by Kelders et al [25] (2.6, 1.6, and 1.3, respectively).

Persuasive design was a significant predictor of effect size in the meta-regression of ICBT for depression. The unstandardized β coefficient (*B*) of 0.04 suggested that for each additional persuasive design principle an intervention uses, one could predict the effect size (Hedges *g*) for that intervention to increase by 0.04, compared with a control condition in a randomized trial. However, meta-regression is an inherently observational procedure [100], and the results therefore could not show whether persuasive design *caused* certain ICBT interventions for depression to be more efficacious than others. Persuasive design did not predict efficacy in the meta-regression of ICBT for anxiety. However, it is worth noting that the meta-regression of ICBT for anxiety included far fewer studies than the meta-regression of ICBT for depression and had limited statistical power to identify an effect. Indeed, persuasive design had an unstandardized β coefficient of 0.03 in the meta-regression of ICBT for anxiety, which—although not statistically significant—was comparable in magnitude with that of the meta-regression of ICBT for depression. The results of the meta-regression of unguided ICBT for anxiety should be interpreted cautiously because assumption tests showed that certain assumptions were unmet. Nonetheless, our results suggest that persuasive design is more closely related to outcomes in interventions for depression than anxiety. Given that persuasive design is purported to motivate engagement in treatment [17] and that lack of motivation is a hallmark of depression, it is possible that persuasive design is particularly important in ICBT for depression.

Overall, our findings support the hypothesis that persuasive design predicts efficacy in unguided ICBT, at least in the treatment of depression. Our findings also support the validity of the PSD framework [17] by showing that it is meaningfully related to treatment outcomes. Although the results do not demonstrate the importance of any specific persuasive design principles, they support the growing body of theory and data suggesting, broadly, that persuasive design matters in eHealth [18-24]. These findings are encouraging and timely. ICBT has become well established over the last two decades, having now been evaluated in hundreds of trials [101] and currently being funded by many governments around the world [102]. It is clear that ICBT is effective, and a natural next step in ICBT research will be to explore possible avenues for making it more effective. Our findings suggest that enhanced persuasive design may be one such avenue. Notably, because ICBT is highly scalable, particularly when it is unguided, even slight increases in effectiveness can have substantial and wide-reaching implications for public health.

### Limitations

This study had several limitations. First, a considerable amount of data was unreported; in particular, it is likely that many interventions used persuasive design principles that were not described in the included studies. Second, although we were able to identify the principles in the PSD framework as present or absent, we did not have access to the interventions themselves, and we were unable to evaluate how effectively persuasive design principles were implemented. Third, we were unable to show, through our meta-regressions, whether specific persuasive design principles predicted efficacy. Finally, only 1 researcher was involved in data extraction; a second extractor would have helped reduce the risk of error, inconsistency, or bias.

### Future Directions

Further research will be required to clarify the role of persuasive design in unguided ICBT and other eHealth interventions. First, dismantling studies comparing versions of interventions with and without certain persuasive design principles could evaluate the utility of specific principles. Factorial randomized trials of this kind would allow researchers to efficiently evaluate multiple persuasive design principles in a single study. Second, it would be helpful to explore how intervention users experience persuasive design, which could perhaps be achieved through qualitative research or the development of a self-report questionnaire assessing user experiences of persuasive design. Third, the literature would benefit from a more detailed description of persuasive design in unguided ICBT interventions based on a careful review of the interventions themselves (ie, rather than this study’s review of descriptions of interventions from randomized trials). Finally, further research will be required to test our finding that persuasive design predicts efficacy in unguided ICBT for depression but not for anxiety.

### Conclusions

The literature on ICBT and other eHealth interventions is evolving rapidly. This review has provided an updated meta-analysis of unguided ICBT for depression and anxiety, generally finding smaller effect sizes for depression than for anxiety. It has also documented the wide variability in the use of persuasive design in unguided ICBT and demonstrated through a meta-regression that persuasive design predicts efficacy in unguided ICBT for depression. Persuasive design is a promising avenue for further optimization of eHealth interventions, including ICBT, and an area of research that is worth investigating further.

## Appendix

Multimedia Appendix 1 The persuasive systems design framework.

Multimedia Appendix 2 Recommendations for making eHealth interventions more engaging and related persuasive design principles.

Multimedia Appendix 3 Log of revisions and clarifications to the original methodological protocol.

Multimedia Appendix 4 Search terms.

Multimedia Appendix 5 Data items.

Multimedia Appendix 6 Flow of studies in the original literature search (October 29, 2019).

Multimedia Appendix 7 Flow of studies in the revised literature search (July 2, 2020).

Multimedia Appendix 8 Assumption tests for meta-regression of unguided internet-delivered cognitive behavioral therapy for depression.

Multimedia Appendix 9 Assumption tests for meta-regression of unguided internet-delivered cognitive behavioral therapy for anxiety.

## Abbreviations

CBT: cognitive behavioral therapy
ICBT: internet-delivered cognitive behavioral therapy
PRISMA: Preferred Reporting Items for Systematic Reviews and Meta-Analyses
PSD: persuasive systems design
