# Supplementary material for: Exploring the Role of Persuasive Design in Unguided Internet-Delivered Cognitive Behavioral Therapy for Depression and Anxiety Among Adults: Systematic Review, Meta-analysis, and Meta-regression
Source: J Med Internet Res. 2021 Apr 29;23(4):e26939. doi: 10.2196/26939 (PMC8120424; doi:10.2196/26939)
Supplement: Multimedia Appendix 1 [file jmir_v23i4e26939_app1.docx]

## Multimedia Appendix 1

**The persuasive systems design framework.**

| Principle | | Description^a^ |
| --- | --- | --- |
|  |  |  |
| **Primary task support** | | |
|  | Reduction | A system that reduces complex behavior into simple tasks helps users perform the target behavior, and it may increase the benefit/cost ratio of a behavior. |
|  | Tunneling | Using the system to guide users through a process or experience provides opportunities to persuade along the way. |
|  | Tailoring | Information provided by the system will be more persuasive if it is tailored to the potential needs, interests, personality, usage context, or other factors relevant to a user group. |
|  | Personalization | A system that offers personalized content or services has a greater capability for persuasion. |
|  | Self-monitoring | A system that keeps track of one’s own performance or status supports the user in achieving goals. |
|  | Simulation | Systems that provide simulations can persuade by enabling users to observe immediately the link between cause and effect. |
|  | Rehearsal | A system providing means with which to rehearse a behavior can enable people to change their attitudes or behavior in the real world. |
| **Dialogue support** | | |
|  | Praise | By offering praise, a system can make users more open to persuasion. |
|  | Rewards | Systems that reward target behaviors may have great persuasive powers. |
|  | Reminders | If a system reminds users of their target behavior, the users will more likely achieve their goals. |
|  | Suggestion | Systems offering fitting suggestions will have greater persuasive powers. |
|  | Similarity | People are more readily persuaded through systems that remind them of themselves in some meaningful way. |
|  | Liking | A system that is visually attractive for its users is likely to be more persuasive. |
|  | Social role | If a system adopts a social role, users will more likely use it for persuasive purposes. |
| **System credibility support** | | |
|  | Trustworthiness | A system that is viewed as trustworthy will have increased powers of persuasion. |
|  | Expertise | A system that is viewed as incorporating expertise will have increased powers of persuasion. |
|  | Surface credibility | People make initial assessments of the system credibility based on a firsthand inspection. |
|  | Real-world feel | A system that highlights people or organization behind its content or services will have more credibility. |
|  | Authority | A system that leverages roles of authority will have enhanced powers of persuasion. |
|  | Third-party endorsements | Third-party endorsements, especially from well-known and respected sources, boost perceptions on system credibility. |
|  | Verifiability | Credibility perceptions will be enhanced if a system makes it easy to verify the accuracy of site content via outside sources. |
| **Social support** | | |
|  | Social learning | A person will be more motivated to perform a target behavior if (s)he can use a system to observe others performing the behavior. |
|  | Social comparison | System users will have a greater motivation to perform the target behavior if they can compare their performance with the performance of others. |
|  | Normative influence | A system can leverage normative influence or peer pressure to increase the likelihood that a person will adopt a target behavior. |
|  | Social facilitation | System users are more likely to perform target behavior if they discern via the system that others are performing the behavior along with them. |
|  | Cooperation | A system can motivate users to adopt a target attitude or behavior by leveraging human beings’ natural drive to co-operate. |
|  | Competition | A system can motivate users to adopt a target attitude or behavior by leveraging human beings’ natural drive to compete. |
|  | Recognition | By offering public recognition for an individual or group, a system can increase the likelihood that a person/group will adopt a target behavior. |

^a^All descriptions were copied verbatim from Oinas-Kukkonen and Harjumaa (p. 492-495) [17].
