# Supplementary material for: Exploring the Role of Persuasive Design in Unguided Internet-Delivered Cognitive Behavioral Therapy for Depression and Anxiety Among Adults: Systematic Review, Meta-analysis, and Meta-regression
Source: J Med Internet Res. 2021 Apr 29;23(4):e26939. doi: 10.2196/26939 (PMC8120424; doi:10.2196/26939)
Supplement: Multimedia Appendix 2 [file jmir_v23i4e26939_app2.docx]

## Multimedia Appendix 2

**Recommendations for making eHealth interventions more engaging and related persuasive design principles.**

| Recommendations | | Related PSD framework principles |
| --- | --- | --- |
|  | |  |
| **Helgadóttir et al., 2009 [20]** | |  |
|  | Automated individualization of content | *Personalization* |
|  | Examples of completing treatment tasks | *Simulation* |
|  | Automated reminders | *Reminders* |
|  | Time-limited access | Not included in PSD framework |
|  | Simulated therapist contact | *Social role* |
|  | Opportunities to repeat treatment tasks | *Rehearsal* |
| **Brouwer et al., 2011 [23]** | |  |
|  | Automated feedback | Various *dialogue support* principles |
|  | Action or activity planning | *Reduction* |
|  | Self-monitoring | *Self-monitoring* |
|  | Feedback on progress | *Self-monitoring* or various *dialogue support* principles |
|  | Supplementary online activities | Not included in PSD framework |
| **Schubart et al., 2011 [21]** | |  |
|  | Individualizing content | *Personalization* |
|  | Targeting specific user population | *Tailoring* |
| **Morrison et al., 2012 [18]** | |  |
|  | Social context and support | Various *social support* principles |
|  | Contact with intervention | Various *dialogue support* principles |
|  | Individualizing content | *Personalization* |
|  | Self-management | *Self-monitoring* |
| **Søgaard Neilson & Wilson, 2019 [24] (elaborating on Morrison et al.)** | |  |
|  | Enhanced layout | Loosely related to *liking* |
|  | Instructional design | *Reduction*, *tunneling*, and *similarity* |
| **Ludden et al., 2015 [22]** | |  |
|  | Automatic individualization of content | *Personalization* |
|  | Personalization of preferences | *Personalization* |
|  | Targeting specific user population | *Tailoring* |
|  | Relaying ambient information | Loosely related to *liking* |
|  | Greater use of metaphors | Not included in PSD framework |
| **Fleming et al., 2016 [19]** | |  |
|  | Engaging target groups in design process | *Tailoring* |
|  | Greater individualization of content | *Personalization* |
|  | Modular approaches | *Personalization* |
|  | Gamification | Includes but is not limited to r*ewards* and *liking* |
|  | Simulated therapist contact | *Social role* |
|  | Automated reminders | *Reminders* |
|  | Automated feedback | Various *dialogue support* principles |
