# Supplementary material for: Exploring the Role of Persuasive Design in Unguided Internet-Delivered Cognitive Behavioral Therapy for Depression and Anxiety Among Adults: Systematic Review, Meta-analysis, and Meta-regression
Source: J Med Internet Res. 2021 Apr 29;23(4):e26939. doi: 10.2196/26939 (PMC8120424; doi:10.2196/26939)
Supplement: Multimedia Appendix 3 [file jmir_v23i4e26939_app3.docx]

## Multimedia Appendix 3

**Log of revisions and clarifications to the original methodological protocol.**

| **Stage/Date** | | **Change or Clarification** |
| --- | --- | --- |
| **Literature search** | | |
|  | 10/25/19 | - The database PsycArticles was included along with Medline and PsycINFO in our search via Ovid. - The search string “AND (random OR RCT) AND (trial OR RCT)” was replaced with “AND (random OR control OR trial OR RCT)”. - The search string “AND (Internet OR web OR online)” was replaced with “AND (Internet OR web OR online OR behavio* intervention technology OR compute* OR cybertherapy OR digital OR e-intervention OR e-mental health OR e-therapy OR eHealth OR e-Health OR tele-therapy OR telehealth)”. |
|  | 10/26/19 | - The search string “(unguided OR self-guided OR self-directed OR standalone)” was removed. - The phrase “OR app” was added to the search string “AND (Internet OR web OR online OR behavio* intervention technology OR compute* OR cybertherapy OR digital OR e-intervention OR e-mental health OR e-therapy OR eHealth OR e-Health OR tele-therapy OR telehealth)”. |
| **Eligibility Screening** | | |
|  | 10/29/19 | - It was decided that studies would not be excluded on the basis of diagnostic interviews being employed (i.e., diagnostic interviews would not be considered guidance). |
|  | 10/31/19 | - It was decided that studies whose samples had a mean age of less than 18 years would be excluded. |
|  | 12/05/19 | - It was decided that the following treatment approaches would be considered CBT:   - Exposure and response prevention   - Problem-solving therapy   - Behavioural activation   - Worry exposure   - Imaginal exposure   - Acceptance and commitment therapy - It was decided that the following treatment approaches would not be considered CBT:   - Attention bias modification training   - Cognitive bias modification   - Bias modification   - Interpretation bias modification   - Written disclosure   - Gratitude intervention |
|  | 12/13/19 | - It was decided that studies would be excluded if their samples had significant health, somatic, psychological, or psychiatric concerns excluding mood- and anxiety-related concerns. For example, a study evaluating an ICBT intervention among cancer patients or problem gamblers would be excluded. |
| **Data Extraction** | | |
|  | 1/14/20 | - It was decided that instead of “using whichever measure is described as the primary outcome measure in each study” to calculate Hedges’ *g*, as per the protocol submitted to Prospero, Hedges’ *g* would be calculated using whichever measure is described as the primary *symptom* measure, as some studies employed primary outcome measures that were not symptom measures. |
|  | 1/16/20 | - It has been argued compellingly that within-groups effect size should not be used as a primary outcome measure in meta-analysis (Cuijpers et al., 2017 and Hoyt & Del Re, 2017). Therefore, during data extraction and prior to any data analysis, it was decided that between-groups effect size would be used as the primary outcome measure instead of within-groups effect size. |
|  | 1/19/20 | - It was decided during data extraction that anxiety-related disorders (e.g., obsessive-compulsive disorder, posttraumatic stress disorder) would not be included in the review. All identified studies of interventions for obsessive compulsive disorder and posttraumatic stress were therefore excluded before any data was extracted from them. This was done in an effort to define the scope of the review more narrowly. |
|  | 2/03/20 | - It was decided that lab-based studies (i.e., studies in which participants completed their assigned interventions in a lab environment) could not be considered unguided, as research personnel were required to be present in such studies and played a guiding role. |
|  | 2/05/20 | - It was clarified that any contact between participants and the research team that was of a therapeutic nature would constitute guidance and would therefore be considered a reason for exclusion of a study. This included cases in which researchers monitored symptoms and extended guidance to participants who experienced increases in symptoms or suicidal ideation. This did not include screening interviews or reminders to use the intervention, so long as those reminders did not appear to be therapeutic in nature. - It was decided that studies would be excluded if their samples had very low scores on symptom measures at baseline. |
|  |  | - It was clarified that an intervention that is not primarily CBT (e.g., an expressive writing intervention with CBT elements) would not be considered ICBT, at the discretion of the eligibility screeners. |
|  | 03/03/20 | - It was clarified that an unguided intervention could be included even if the research team provided some encouragement to participants to complete an intervention, so long as the following conditions were met:   - Contact from the research team was of a manner that *could* be automated, such as standardized emails. Studies in which the research team called participants by phone to encourage use of the intervention were excluded.   - Contact from the research team was clearly not therapeutic in nature. Note that studies in which a subset of participants (e.g., participants in crisis) received therapeutic contact from the research team were excluded. |
| **Data Analysis** | | |
|  | 03/05/20 | - In the original protocol, it was written that an intention-to-treat approach would be used for handling missing data. This is not possible without individual participant data, so an intention-to-treat approach to handling missing data was not used. |
|  | 03/05/20 | - The original protocol described a number of analyses related to completion rate. However, completion rate was reported in only a handful of cases, so these analyses were not feasible. Completion rate was therefore not assessed in any analyses. |
|  | 03/20/20 | - Due to uncertainty surrounding the procedure for calculating a 95% confidence interval around the *I*^2^ statistic, the significance of heterogeneity was determined using the *Q* statistic instead. The *I*^2^ statistic was still calculated for descriptive purposes. |
|  | 03/22/20 | - Egger’s test was not used to determine whether publication bias was statistically significant because the technique was not available in Comprehensive Meta Analysis. Funnel plots and Duval and Tweedie’s trim and fill technique were still employed. |
|  | 06/17/20 | - Many researchers have recommended minimum numbers of subjects per variables in regression analyses. According to Austin and Steyerberg (2015), various research groups have recommended five, 10, or 15-20 subjects per variable. To avoid overfitting the regression models, meta-regressions were conducted using only three predictors in the depression meta-regression (total number of persuasive design principles, target symptoms, control condition type) and only the first two of these predictors in the anxiety meta-regression. |
