# Supplementary material for: Exploring the Role of Persuasive Design in Unguided Internet-Delivered Cognitive Behavioral Therapy for Depression and Anxiety Among Adults: Systematic Review, Meta-analysis, and Meta-regression
Source: J Med Internet Res. 2021 Apr 29;23(4):e26939. doi: 10.2196/26939 (PMC8120424; doi:10.2196/26939)
Supplement: Multimedia Appendix 4 [file jmir_v23i4e26939_app4.docx]

## Multimedia Appendix 4

**Search terms.**

Note that the eligibility criteria were revised after the search was conducted. Therefore, some terms (e.g., “OCD”, “PTSD”) were not relevant. Please see Multimedia Appendix 3 for details of revisions to the original methodological protocol.

| **Ovid Search (PsycINFO, Medline, & PsycArticles)**  ((CBT or cognitive behavio* therapy) and (Internet or web or online or behavio* intervention technology or compute* or cybertherapy or digital or e-intervention or e-mental health or e-therapy or eHealth or e-Health or tele-therapy or telehealth or app) and (random or control or trial or RCT) and (depression or low mood or anxiety or GAD or SAD or panic or PD or agoraphobia or selective mutism or phobia or obsessive-compulsive or OCD or acute stress or post-traumatic stress or PTSD)).ab,kw,ti.  *Filters applied separately to limit search to articles published in English since 2000.*  **PubMed Search**  ((((CBT[Title/Abstract] OR cognitive behavio* therapy[Title/Abstract]) AND (Internet[Title/Abstract] OR web[Title/Abstract] OR online[Title/Abstract] OR behavio* intervention technology[Title/Abstract] OR compute*[Title/Abstract] OR cybertherapy[Title/Abstract] OR digital[Title/Abstract] OR e-intervention[Title/Abstract] OR e-mental health[Title/Abstract] OR e-therapy[Title/Abstract] OR eHealth[Title/Abstract] OR e-Health[Title/Abstract] OR tele-therapy[Title/Abstract] OR telehealth[Title/Abstract] OR app[Title/Abstract]) AND (random[Title/Abstract] OR control[Title/Abstract] OR trial[Title/Abstract] OR RCT[Title/Abstract]) AND (depression[Title/Abstract] OR low mood[Title/Abstract] OR anxiety[Title/Abstract] OR GAD[Title/Abstract] OR SAD[Title/Abstract] OR panic[Title/Abstract] OR PD[Title/Abstract] OR agoraphobia[Title/Abstract] OR selective mutism[Title/Abstract] OR phobia[Title/Abstract] OR obsessive-compulsive[Title/Abstract] OR OCD[Title/Abstract] OR acute stress[Title/Abstract] OR post-traumatic stress[Title/Abstract] OR PTSD[Title/Abstract]))) AND Journal Article[ptyp] AND ( "2000/01/01"[PDat] : "2019/12/31"[PDat] ) AND English[lang])  **Web of Science Search**  TS=((CBT OR cognitive behavio* therapy) AND (Internet OR web OR online OR behavio* intervention technology OR compute* OR cybertherapy OR digital OR e-intervention OR e-mental health OR e-therapy OR eHealth OR e-Health OR tele-therapy OR telehealth OR app) AND (random OR control OR trial OR RCT) AND (depression OR low mood OR anxiety OR GAD OR SAD OR panic OR PD OR agoraphobia OR selective mutism OR phobia OR obsessive-compulsive OR OCD OR acute stress OR post-traumatic stress OR PTSD))  *Filters applied separately to limit search to articles published in English since 2000.* |
| --- |
