# Supplementary material for: Exploring the Role of Persuasive Design in Unguided Internet-Delivered Cognitive Behavioral Therapy for Depression and Anxiety Among Adults: Systematic Review, Meta-analysis, and Meta-regression
Source: J Med Internet Res. 2021 Apr 29;23(4):e26939. doi: 10.2196/26939 (PMC8120424; doi:10.2196/26939)
Supplement: Multimedia Appendix 5 [file jmir_v23i4e26939_app5.docx]

## Multimedia Appendix 5

**Data items.**

| Study Characteristics | Study name |
| --- | --- |
|  | Outcome measure(s) |
|  | Article ID |
|  | Year |
|  | Recruitment source |
|  | Recruitment method |
|  | Cost/payment |
|  | Screening procedure |
|  | Pre-post time |
|  | Control condition type |
|  | Researcher contact |
| Risk of Bias | Random sequence generation |
|  | Allocation concealment |
|  | Selective reporting |
|  | Other bias |
|  | Attrition bias (due to incomplete outcome data) |
| General Intervention Characteristics | Name of intervention |
|  | Intervention number |
|  | Target disorder/symptoms |
|  | Theoretical orientation |
|  | Number of modules |
|  | Mode of delivery (e.g., web browser) |
|  | Method of delivery |
| PSD Framework Principles—Primary Task Support | Reduction |
|  | Tunneling |
|  | Tailoring |
|  | Personalization |
|  | Self-monitoring |
|  | Simulation |
|  | Rehearsal |
| PSD Framework Principles—Dialogue Support | Praise |
|  | Rewards |
|  | Reminders |
|  | Suggestion |
|  | Similarity |
|  | Liking |
|  | Social role |
| PSD Framework Principles—Social Support | Social learning |
|  | Social comparison |
|  | Normative influence |
|  | Social facilitation |
|  | Cooperation |
|  | Competition |
|  | Recognition |
| Miscellaneous notes | Miscellaneous notes |
| Outcome data | (Varied from one study to another) |
