# Supplementary figures and images for: Exploring the Role of Persuasive Design in Unguided Internet-Delivered Cognitive Behavioral Therapy for Depression and Anxiety Among Adults: Systematic Review, Meta-analysis, and Meta-regression
Source: J Med Internet Res. 2021 Apr 29;23(4):e26939. doi: 10.2196/26939 (PMC8120424; doi:10.2196/26939)

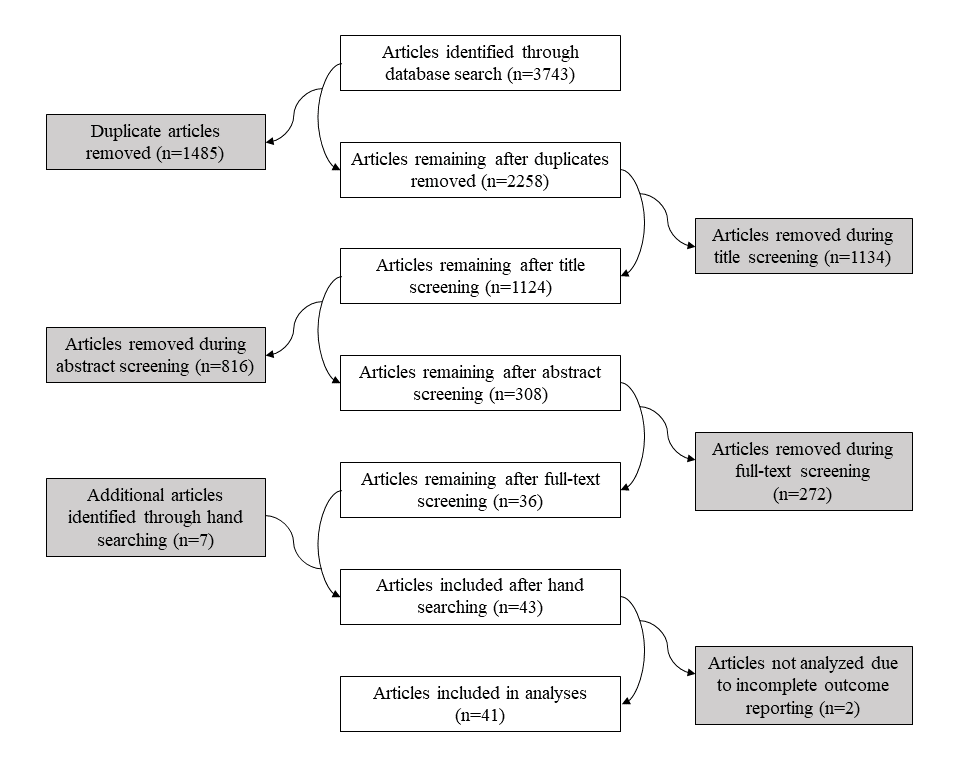

Supplement: Multimedia Appendix 6 [file jmir_v23i4e26939_app6.png]

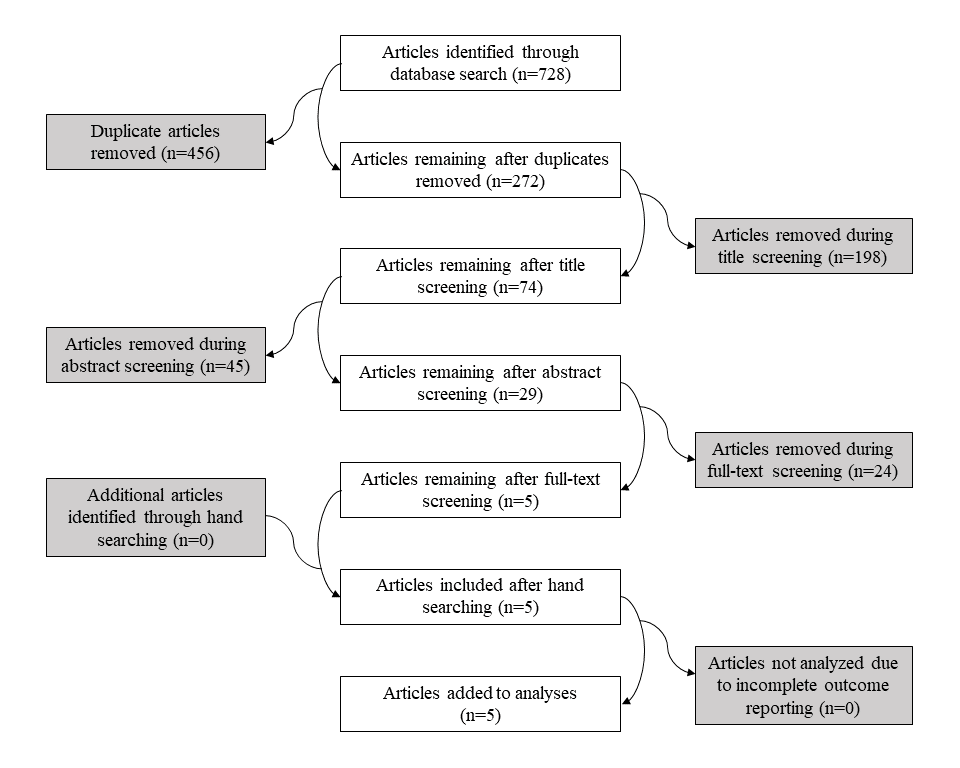

Supplement: Multimedia Appendix 7 [file jmir_v23i4e26939_app7.png]
