# Supplementary material for: Exploring the Role of Persuasive Design in Unguided Internet-Delivered Cognitive Behavioral Therapy for Depression and Anxiety Among Adults: Systematic Review, Meta-analysis, and Meta-regression
Source: J Med Internet Res. 2021 Apr 29;23(4):e26939. doi: 10.2196/26939 (PMC8120424; doi:10.2196/26939)

## Multimedia Appendix 8

**Assumptional tests for meta-regression of unguided ICBT for depression.**

| Step/Assumption | | Description |
| --- | --- | --- |
| Step 1 | | Predicting Hedges’ *g* with whether an intervention was designed to treat anxiety in addition to depression and whether a study employed an active control condition, as opposed to a passive (e.g., waitlist) control condition. |
|  | Linearity | The assumption was met. It would not be possible to find nonlinear relationships between the two predictors in this step and Hedges’ *g* because both predictors were dichotomous. |
|  | No unduly influential outliers | The assumption was met. The highest Cook’s distance value was 0.15. |
|  | Normality of residuals | The assumption was met. Studentized residuals approximated a normal distribution (see Figure 1). |
|  | Homoscedasticity of residuals | The assumption was met or violated only slightly. Studentized residuals appeared to be slightly closer to zero for lesser predicted effect sizes but were comparable across the range of predicted effect sizes (see Figure 2). |
|  | Minimal multicollinearity | The assumption was met. Variance inflation factors for both predictors in this step were 1.11. |
| Step 2 | | Predicting Hedges’ *g* with three variables: whether an intervention was designed to treat anxiety in addition to depression; whether a study employed an active control condition, as opposed to a passive (e.g., waitlist) control condition; and the total number of persuasive design principles identified for each intervention. |
|  | Linearity | The assumption was met. It would not be possible to identify nonlinear relationships between Hedges’ *g* and the two dichotomous predictors in this step (i.e., whether each study employed an active control condition and whether each intervention was designed to treat symptoms of anxiety in addition to depression). Hedges’ *g* had a small to moderate but non-significant correlation with the number of persuasive design principles identified for each intervention, *r*(35) = .28, *P* = .09. We inspected a scatterplot to assess for a possible nonlinear relationship between Hedges’ *g* and number of persuasive design principles. The relationship appeared linear (see Figure 3). |
|  | No unduly influential outliers | The assumption was met. The highest Cook’s distance value was 0.31. |
|  | Normality of residuals | The assumption was met. Studentized residuals approximated a normal distribution (see Figure 4). |
|  | Homoscedasticity of residuals | The assumption was met or violated only slightly. Studentized residuals appeared to be slightly closer to zero for greater predicted effect sizes but were comparable across the range of predicted effect sizes (see Figure 5). |
|  | Minimal multicollinearity | The assumption was met. The highest variance inflation factor for any predictor in this step was 1.36. |

*Note*. Comprehensive Meta-Analysis provided Cook’s distance values and variance inflation factors to test assumptions related to outliers and multicollinearity. The remaining three assumptions were tested in SPSS using the default weights provided by Comprehensive Meta-Analysis (i.e., weighting each study by the inverse of the within-study variance of the primary outcome measure plus the between-study variance.

Figure 1. Distribution of studentized residuals at step 1.

**
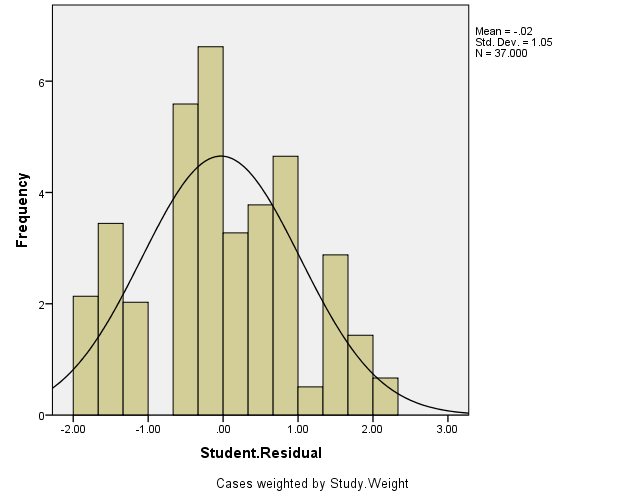
**

Figure 2. Relationship between studentized residuals and predicted values at step 1.

**
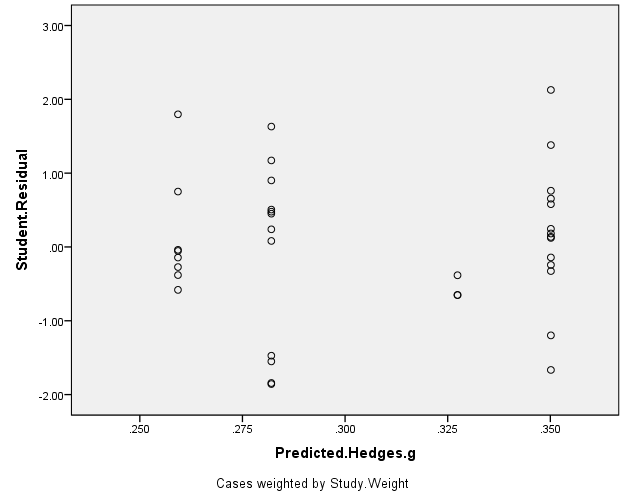
**

Figure 3. Relationship between Hedges’ g and persuasive design principles at step 2.


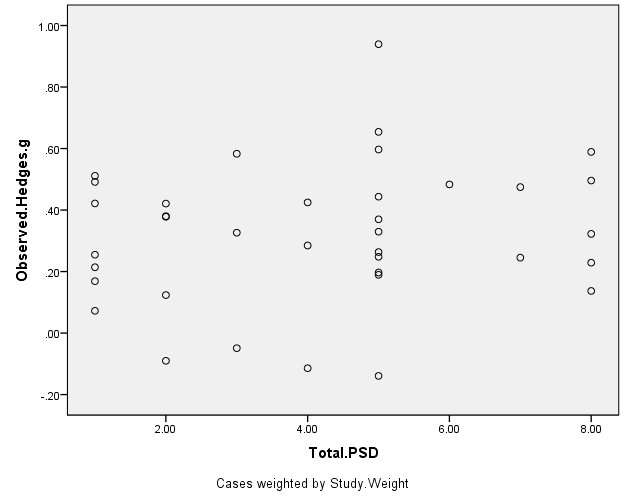


Figure 4. Distribution of studentized residuals at step 2.


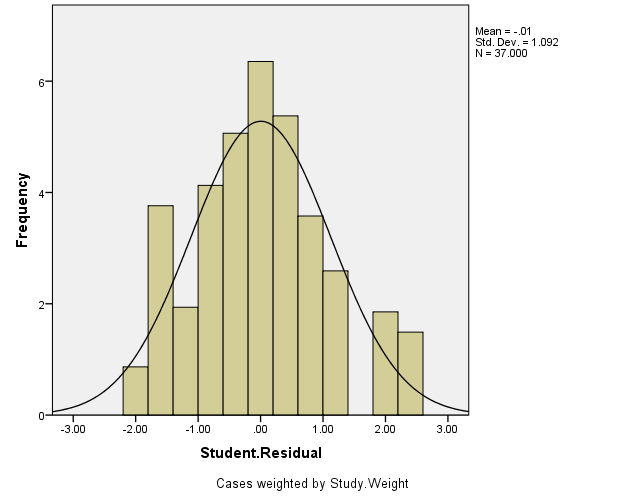


Figure 5. Relationship between studentized residuals and predicted effect size at step 2.


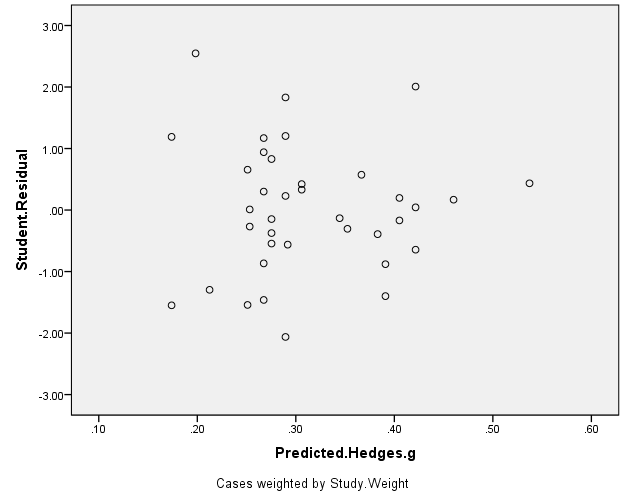

Supplement: Multimedia Appendix 8 [file jmir_v23i4e26939_app8.docx]
