# Supplementary material for: Exploring the Role of Persuasive Design in Unguided Internet-Delivered Cognitive Behavioral Therapy for Depression and Anxiety Among Adults: Systematic Review, Meta-analysis, and Meta-regression
Source: J Med Internet Res. 2021 Apr 29;23(4):e26939. doi: 10.2196/26939 (PMC8120424; doi:10.2196/26939)

## Multimedia Appendix 9

**Assumptional tests for meta-regression of unguided ICBT for anxiety.**

| Step/Assumption | | Description |
| --- | --- | --- |
| Step 1 | | Predicting Hedges’ *g* with whether an intervention was designed to treat depression in addition to anxiety |
|  | Linearity | The assumption was met. Hedges’ *g* was moderately negatively correlated with whether each intervention targeted symptoms of depression, *r*(19) = -.49, *P* = .02. A scatterplot was not inspected to assess for a potential nonlinear relationship because the only predictor in this step was dichotomous. |
|  | No unduly influential outliers | The assumption was met. The highest Cook’s distance value was 0.34. |
|  | Normality of residuals | The assumption may have been violated slightly. Studentized residuals only roughly approximated a normal distribution (see Figure 1). |
|  | Homoscedasticity of residuals | The assumption was not met. Studentized residuals were closer to zero for lower predicted values (i.e., for cases in which the intervention targeted symptoms of depression; see Figure 2). |
|  | Minimal multicollinearity | The assumption could not be violated, because there was only one predictor variable. |
| Step 2 | | Predicting Hedges’ *g* with whether an intervention was designed to treat depression in addition to anxiety and the total number of persuasive design principles identified for each intervention. |
|  | Linearity | The assumption was met. Hedges’ *g* could not have a nonlinear relationship with whether each intervention targeted symptoms of depression because this variable was dichotomous. Hedges’ *g* was moderately correlated with number of persuasive design principles identified, *r*(19) = .40, *P* = .08. We inspected a scatterplot to check for a nonlinear relationship between Hedges’ *g* and number of persuasive design principles, but the relationship appeared linear (see Figure 3). |
|  | No unduly influential outliers | The assumption was met. The highest Cook’s distance value was 0.37. |
|  | Normality of residuals | The assumption may have been violated somewhat. Studentized residuals only roughly approximated a normal distribution (see Figure 4). |
|  | Homoscedasticity of residuals | The assumption was met. Studentized residuals varied similarly across the range of observed effect sizes (see Figure 5). |
|  | Minimal multicollinearity | The assumption was met. Variance inflation factors for both predictors in this step were 1.07. |

*Note*. Comprehensive Meta-Analysis provided Cook’s distance values and variance inflation factors to test assumptions related to outliers and multicollinearity. The remaining three assumptions were tested in SPSS using the default weights provided by Comprehensive Meta-Analysis (i.e., weighting each study by the inverse of the within-study variance of the primary outcome measure plus the between-study variance).

Figure 1. Distribution of studentized residuals at step 1.


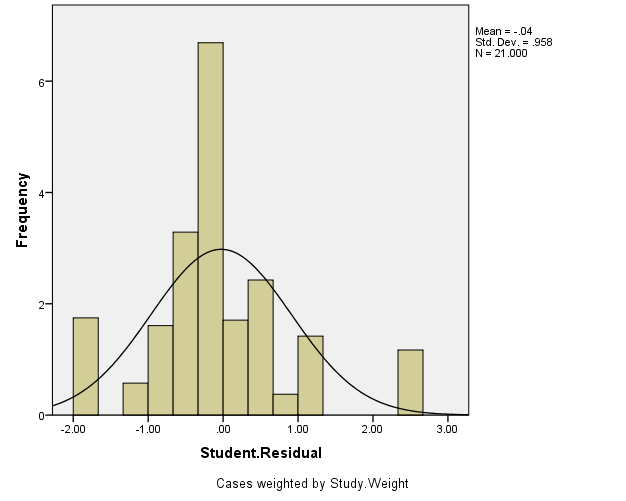


Figure 2. Relationship between studentized residuals and predicted values at step 1.

**
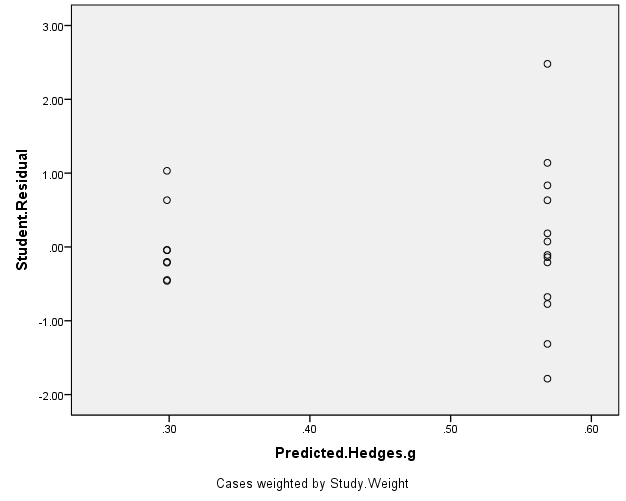
**

Figure 3. Relationship between Hedges’ g and persuasive design principles at step 2.


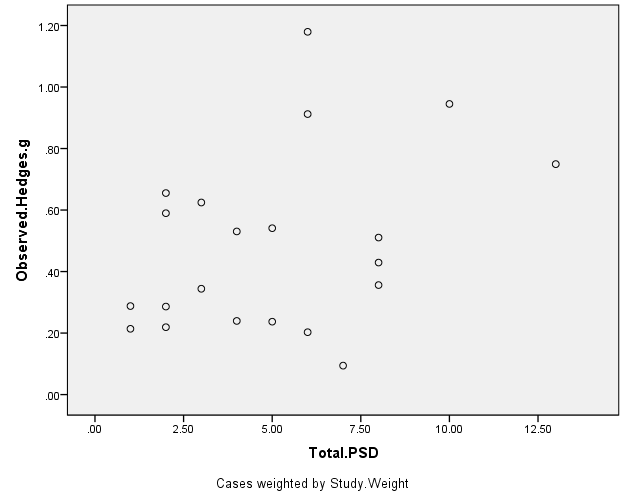


Figure 4. Distribution of studentized residuals at step 2.

*
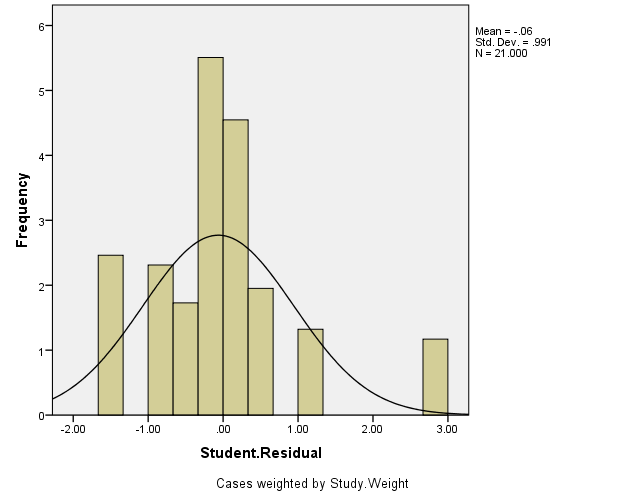
*

Figure 5. Relationship between studentized residuals and predicted effect size at step 2.


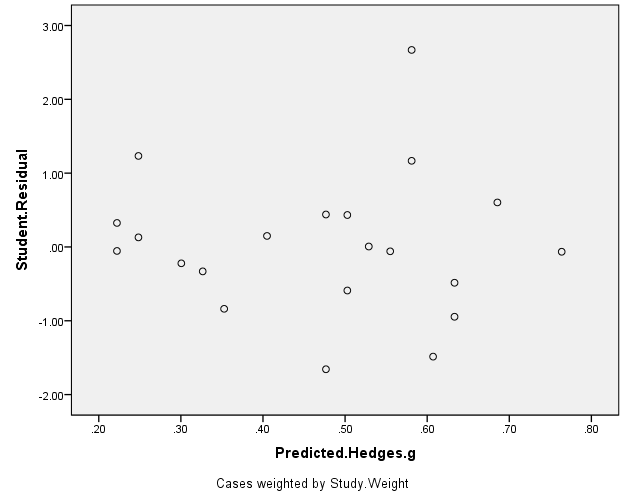

Supplement: Multimedia Appendix 9 [file jmir_v23i4e26939_app9.docx]
